# Interleukin (IL)‐33: an orchestrator of immunity from host defence to tissue homeostasis

**DOI:** 10.1002/cti2.1146

**Published:** 2020-06-17

**Authors:** Zekun Zhou, Fei Yan, Ousheng Liu

**Affiliations:** ^1^ Hunan Key Laboratory of Oral Health Research & Hunan 3D Printing Engineering Research Center of Oral Care & Hunan Clinical Research Center of Oral Major Diseases and Oral Health & Xiangya Stomatological Hospital & Xiangya School of Stomatology Central South University Changsha Hunan China

**Keywords:** immune cells, immune regulation, interleukin‐33, ST2 expression, tissue homeostasis

## Abstract

Interleukin (IL)‐33, a member of the IL‐1 superfamily, functions as an alarm signal, which is released upon cell injury or tissue damage to alert the immune system. It has emerged as a chief orchestrator in immunity and has a broad pleiotropic action that influences differentiation, maintenance and function of various immune cell types via the ST2 receptor. Although it has been strongly associated with immunopathology, critically, IL‐33 is involved in host defence, tissue repair and homeostasis. In this review, we provide an overview of the signalling pathway of IL‐33 and highlight its regulatory functions in immune cells. Furthermore, we attempt a broader discussion of the emerging functions of IL‐33 in host defence, tissue repair, metabolism, inflammatory disease and cancer, suggesting potential avenues to manoeuvre IL‐33/ST2 signalling as treatment options.

## Graphical abstract

In this article, we summarise the current understanding of IL‐33 and its effect on immune cells and focus on the functions of IL‐33 in host defence and inflammation. In particular, we highlight their less well‐appreciated roles in tissue repair and homeostasis.

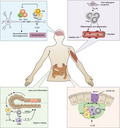

## Introduction

Interleukin (IL)‐33 was identified as a cytokine member of the IL‐1 family in 2005 by GenBank databases.^1^ IL‐33 protein is constitutively and extensively present in healthy mice and humans, and is primarily stored in the nucleus of non‐haematopoietic cells,^1^ including epithelial cells, endothelial cells and keratinocytes, particularly in tissue barrier sites and fibroblastic reticular cells (FRCs) of lymph nodes, as well as mesenchymal cells.^1^, ^2^, ^3^, ^4^, ^5^ It has been proposed that IL‐33 was described as an alarmin that is stored in the nucleus and functioned as an extracellular cytokine in its full‐length form (amino acid 1‐270) when released in response to cell or tissue damage.^3^ Full‐length IL‐33 (IL‐33_FL_) is biologically active, but proteases derived from different cellular sources, such as neutrophils and mast cells, process bioactive IL‐33_FL_ into N‐terminally truncated forms (IL‐33_95–270_, IL‐33_99–270_, IL‐33_107–270_, IL‐33_109–270_ and IL‐33_111‐270_) that have up to 30‐fold higher biological activity than IL‐33_FL_.^3^, ^6^ Another protein ST2, also called T1 or IL‐1 receptor‐like 1 (IL1RL1), is the only well‐documented receptor for IL‐33. Upon ligand binding, the receptor‐associated kinase undergoes recruitment and phosphorylation, which exerts IL‐33 cytokine activity and leads to a series of downstream reactions.^1^ Primarily, IL‐33 was associated with type 2 immune response,^7^, ^8^ accompanied by robust production of IL‐5 and IL‐13.^1^ Expression of ST2 was discovered in dendritic cells (DCs), mast cells (MCs), basophils, eosinophils, neutrophils, macrophages, group 2 innate lymphoid cells (ILC2s), T helper (Th) 2 cells, B cells and regulatory T cells (Tregs).^9^ However, recent studies show that IL‐33 also drives type 1 immune response^10^, ^11^ and plays a protective antiviral response.^11^, ^12^ Natural killer (NK) cells, NKT cells, CD8^+^ T cells and particularly Th1 cells were shown to feature ST2 expression.^4^, ^11^, ^13^ This pleiotropic nature of IL‐33 and unique ST2 expression likely explains why IL‐33 participates in infection, inflammation, tissue homeostasis and repair within these immune cellular networks.^5^, ^12^ Despite this pleiotropic spectrum, important questions remain regarding how IL‐33 acts on different immune cell populations during Th1 and Th2 cell‐mediated immunity, and these questions require clarification. Within this review, we have summarised the current understanding of IL‐33 and its mode of action in immune cells. Although recent reviews have mostly focused on the functions of IL‐33 in inflammatory diseases, we also highlight their less well‐appreciated roles in tissue repair and homeostasis. In this regard, we believe that IL‐33 is essential for furthering endeavours towards its clinical applications.

## IL‐33 receptor and signal transductions

The crystal structure of the ectodomain of ST2 complexed with IL‐33 has been determined.^3^ The ectodomain of ST2 contains an extracellular domain, which binds IL‐33 with the help of IL‐1 receptor accessory protein (IL‐1RAP) to initiate signalling. The binary IL‐33‐ST2 leads to the recruitment of MyD88 and IL‐1R‐associated kinase (IRAK). This complex then activates downstream signalling of mitogen‐activated protein kinases (MAPK), Erk1/2, p38, c‐Jun N‐terminal kinases (JNK) and NF‐κB through TRAF6.^1^, ^3^ IL‐33 elicits cytokine production and other cellular effects by these signals. For example, IL‐13 production results from p38‐MK2/3 activation in DCs stimulated with IL‐33.^14^ By activating JNK and NF‐κB, IL‐33 directly induces type 2 production in neutrophils.^15^

ST2 is broadly expressed on the cell surface of various haematopoietic cells, and it has been documented that ST2 is the only receptor of IL‐33. The unique biologic effect of IL‐33 is mediated by ST2 expression, which can explain the orchestrator function of IL‐33. Michael Peine *et al*. detail evidence indicating that IL‐33 enhances the expression of the lineage‐specifying transcription factors for T cells. In turn, these transcription factors control and enhance ST2 expression, thus triggering a positive feedback mechanism. The Th1 lineage‐specifying transcription factors, T‐bet and STAT4, are required for ST2 expression. IL‐33 supports the upregulation of T‐bet and STAT4, thus fuelling a positive feedback loop that strengthens Th1 cell differentiation via its own receptor, ST2.^9^, ^11^ Taken together, IL‐33 may control effector function in haematopoietic cells through ST2 expression.

## IL‐33‐responsive immune cell

IL‐33 is an alarmin that strongly promotes the activity of the immune system.^3^ Principally, it was associated with type 2 innate and adaptive immunity and inflammation, characterised by the production of IL‐4, IL‐5 and IL‐13.^1^, ^7^ Hence, for a deep understanding of the IL‐33 system, it is necessary to know^12^ how these ST2‐expressing cell types orchestrate the response to a constitutive or inducible IL‐33 cytokine.

### Dendritic cells

Dendritic cells express ST2 and respond directly to IL‐33.^14^, ^16^ Under treatment of IL‐33, DCs can be activated to release cytokines.^14^ Cell‐surface expression of OX40L, MHC class II and CD86 is upregulated,^16^ which is associated with DC and T‐cell interaction. It has long been established that antigen presentation by DCs is crucial to bridge innate and adaptive immunity, primarily their direction of Th cell differentiation.^16^ In the presence of DCs, IL‐33 can direct naive CD4^+^ T cells towards Th2 cells^16^, ^17^ and promote its proliferation and Th2 cytokine production, whether the antigen presentation and recognition exists or not.^18^ Although the DC subset is involved in allergy and parasite reaction by polarising and initiating Th2 cell immunity, its mechanism has not been identified yet. In fact, several studies have indicated that the expression of functional surface molecules in DC subsets dictates Th2 differentiation. For example, on the one hand IL‐33‐stimulated DCs express upregulated OX40L and promote Th2 cell‐mediated immunity.^16^, ^19^ On the other hand, PPAR‐γ and IRF4 also control the capacity of DCs to polarise Th2 cells and are critical for IL‐33‐stimulated Th2 effector function.^17^, ^20^ Curiously, IL‐10 and IL‐17 are also detected within the DC cytokine production.^16^ Some works describe that IL‐33 is involved in Th17‐bias differentiation and upregulation of Th17‐associated molecule expression of RORγt and IL‐17 by DCs.^16^ By contrast, IL‐33 also supports Treg expansion when exposed to DCs, and ST2^−/−^ DCs fail to promote Treg proliferation.^19^, ^21^ Thus, DCs may maintain the capacity of T cells to differentiate into Th2, Th17 and Treg, and detailed mechanisms remain to be elucidated.

### Mast cells

ST2 mRNA and protein have been detected in MCs,^2^ and IL‐33 was found to activate and expand MCs.^2^, ^22^ When activated, MCs have the capacity to induce high levels of cytokine production, including IL‐5, IL‐6, IL‐13, TNF, IL‐2 and TGF‐β.^2^, ^23^, ^24^ Furthermore, IL‐33 can enhance the expression of the chemokines CCL2, CCL3 and CCL7, which are chemoattractant for macrophages.^2^ CCR7 induces MCs to migrate towards T‐cell‐rich tertiary lymphoid structures (TLSs).^23^ The above effects are mediated by IL‐33 in the absence of IgE crosslinking. Accordingly, IL‐33 significantly augments cytokine production, mediators and even the degranulation magnitude in an IgE‐dependent manner during airway inflammation.^25^, ^26^ In addition to effector function, IL‐33 treatment prolongs the survival of MCs, which is mediated primarily by the upregulation of anti‐apoptotic factor BCLXL.^27^

### Granulocytes

ST2 was reportedly expressed in granulocytes, including eosinophils, neutrophils and basophils.^28^, ^29^, ^30^ These cells have been shown to respond to IL‐33 with an induced overlapping set of cytokine and chemokine productions,^28^ as well as granulation upon IgE triggering. However, in eosinophils, IL‐33 potently enhances its development and superoxide production, as well as survival.^28^, ^29^ Infiltration of robust tissue eosinophils is markedly noted in mice treated with IL‐33.^1^, ^31^, ^32^ Curiously, IL‐33 shaped lung‐resident basophils into a specific gene profile. Subsequently, the basophil polarises the macrophage towards an anti‐inflammatory alternatively activated macrophage (AAM), which maintains a lung‐specific microenvironment.^29^

Information regarding the responsiveness of neutrophils to IL‐33 is limited. Its surface expression was robustly increased when neutrophils were exposed to IL‐33.^30^ Neutrophils are primarily involved in Th2 immune disease, including asthma and infectious diseases. Recent studies showed that IL‐33 can polarise neutrophils and display a distinct gene expression profile. This expression profile of Th2 cytokines includes IL‐4, IL‐5, IL‐9 and IL‐13, suggesting the pathogenesis of IL‐33‐induced neutrophils in airway allergic disease. Furthermore, IL‐33 promotes the synthesis of chemokines CXCL2, CXCL3, CCL7, CCL2 and CXCR2,^15^, ^30^ which promote the influx of neutrophils to the pathogenic site. In this study, phagocytosis and neutrophil killing activity are markedly elevated in mice infected with *Candida albicans*.^33^ Sepsis‐induced mice with ST2‐deleted neutrophils had impaired neutrophil migration ability and had decreased length of survival.^30^

### Macrophages

Macrophages have been reported to constitutively express ST2,^34^ supporting the idea that IL‐33 can stimulate and polarise macrophages towards AAM, dependent of IL‐13 or IL‐4. This has shown a display in upregulated AAM markers, including arginase 1 and Ym1,^33^, ^35^, ^36^ and is accompanied by chemokine production, which may further recruit inflammatory cells.^37^ In this regard, TGF‐β was produced by AAM in bleomycin‐induced fibrosis.^38^ In particular, brain‐derived macrophages have a phagocytic capacity when exposed to IL‐33 and can also act neuroprotectively.^39^ Furthermore, IL‐33 promotes macrophages to express CCR2 and recruit macrophages to the site of injury.^39^ By contrast, IL‐33 regulates the functions of macrophages through a Toll‐like receptor. When responding to LPS, IL‐33‐primed macrophages express increased levels of MD2 and TLR‐4, thereby elevating its pro‐inflammatory effect.^34^

### NK and iNKT cells

ST2 can also be found on NK and iNKT cells.^4^, ^13^ IL‐33 serves as a potent stimulator of NK and NKT cells. IL‐33 expands NK cell and IFN‐γ production only upon TCR engagement, or in the presence of cytokine IL‐12 *in vitro*.^4^, ^10^, ^13^ Indeed, ST2‐deficient mice showed significantly less virus‐induced IFN‐γ secretion by NK cells when compared to WT mice.^10^ Like NK cells, iNKT cells stimulated by IL‐33 have the ability to proliferate and produce IFN‐γ and IL‐17A.^4^ In contrast, IL‐33‐deficient mice showed impaired recruitment, activation and cytokine production of iNKT cells during ischaemia–reperfusion injury.^4^ However, augmented levels of Th2 cytokines (IL‐4, IL‐5 and IL‐13) were also detected in iNKT cells.^13^

### ILC2s

A heterogeneous group of ILCs are capable of producing large numbers of Th2 cytokines and constitutively expressing ST2.^5^, ^40^, ^41^ This group is collectively termed ILC2s. Based on studies of tissue‐resident ILC2s obtained from tissue, including skin, lung, gastrointestinal tract, islet, brain and adipose tissue, it was proposed that IL‐33 was a potent stimulus for ILC2s.^8^, ^16^, ^41^, ^42^ When activated by IL‐33, ILC2s can release abundant quantities of type 2 cytokines such as IL‐5, IL‐6, IL‐13 and GMSF. In addition, ILC2s can release epithelial growth factors such as amphiregulin (AREG).^5^, ^43^ IL‐33 also increases the migratory capacity of ILC2s via the induction of chemoattractants such as CCXL16 and CCL25.^7^ It supports the fact that ILC2s can home to specific tissue, which contributes to pathological changes in asthma.^7^

However, IL‐33 promotes the development and maintenance of ILC2s. Transcription factors that drive the development of ILC2s such as Gfi1, GATA3 and RORα also participate in the action of IL‐33. The responsiveness of ILC2s to IL‐33 was found to be controlled by Gfi1, which regulates ST2 expression at the surface of ILC2s.^8^ Upon administration of IL‐33, the expression of GATA3 and RORα is upregulated in ILC2s,^8^ and is accompanied by ILC2 expansion.^2^, ^7^ The number of ILC2s was severely compromised in IL‐33‐deficient mice in islets when compared to control mice.^5^ Further, the idea that IL‐33‐induced proliferation in comparison with WT control has been validated using ILC2‐reconstituted *Rag2‐Il2rg*‐ double knockout mice.^5^ Specifically, signalling molecules such as OX40L, ICOSL and PD‐L1 on the cell surface also influence cross‐dialogue between ILC2s and T cells following IL‐33 administration. For example, IL‐33‐induced OX40L in ILC2s from the lungs can mediate adaptive type 2 immunity via OX40 in Th2 cells and Tregs.^19^ Murine ILC2s promote Treg accumulation depending on the interactions between ICOS in Tregs and ICOSL in ILC2s.^42^ The upregulation of PD‐L1 on ILC2s in response to IL‐33 can control Th2 differentiation. This differentiation occurs via dialogue with PD‐1 on CD4^+^ T cells, and thus can promote anti‐helminth infection capabilities.^44^ More specially, IL‐33‐primed ILC2s appear to be more potent in innate immunity, rather than in adaptive immunity. ILC2s are sufficient in experimental models of allergic asthma in order to potentiate IL‐33‐induced airway inflammation and promote AHR.^8^, ^24^

### Th2 cells

ST2 is constitutively expressed at high levels from activated Th2 cells, but not from naive CD4^+^ T cells.^9^ Th2 cells can respond directly to IL‐33 and can express differential and functional programmes. *In vitro* polarisation studies have implicated that IL‐33 can polarise naive CD4^+^ T cells towards activated Th2 cells in order to produce IL‐5 and IL‐13. However, this response only occurs in the presence of TCR triggering.^1^, ^45^ Furthermore, antigen‐specific ST2^+^ Th2 cells were shown to produce more IL‐5 and IL‐13 than the non‐antigen‐specific Th cells and ST2^−/−^ Th2 cells.^46^ Expression of PPAR‐γ on Th2 cells is critical for ST2, which mediates Th2 response in allergic and anti‐infection responses.^47^ During infection with *Nippostrongylus brasiliensis*, intestinal adult worm burden was significantly lower in mice that had received OT‐II Th2 cells in *Rag2^−/−^Il2rg^−/−^* mice than in control mice that did not receive Th2 cells.^45^

### Th17 cells

Information regarding the responsiveness of Th17 cells to IL‐33 is sparse. Recent studies showed that ST2 is expressed in Th17 cells both in mice and in humans.^48^ However, the process by which Th17 cells upregulate ST2 remains unclear. Surprisingly, IL‐33 is capable of inducing the cell suppressor function of Th17 and dampening inflammatory pathology via secretion of IL‐10 in the small intestine.^48^

### Th1 cells

Several published reports have shown that activated Th1 cells express ST2, albeit at relatively lower levels than Th2 cells.^11^ This supports the possibility that IL‐33 acts on Th1. Transcriptional network analyses were used to identify the mechanism underlying ST2 expression of Th1 cells. T‐bet and STAT4, Th1 lineage‐specifying transcription factors, are required for optimal ST2 expression.^9^, ^11^ IL‐33 enhances the expression of transcription factors T‐bet and STAT4, which in turn promotes the expression of ST2. Thus, IL‐33 promotes the differentiation programmes of Th1.^9^ Studies of mice infected with lymphocytic choriomeningitis virus (LCMV) indicated that IL‐33 promotes expansion and cytokine production of Th1 cells, thereby perpetuating antiviral effects.^11^ Furthermore, recent studies demonstrated that IL‐33 can potentiate the action of IL‐12 in Th1 cells, resulting in Th1 cell polarisation and elevated production of IFN‐γ.^49^ However, it remains unclear whether IL‐33 signalling can control functional behaviour and what the mechanism is behind it.

### B cells

IL‑33 has a known role in the development of B‐cell immunoglobulin response. B cells can be effectively activated by IL‐33 stimulation and induce IgA and IgE production in naive mice. This can also augment IgM secretions in inflammatory or allergic disease.^20^, ^50^ In ST2*^−/−^* mice with transferred IL‐33‐activated B1 cells, contact sensitivity was significantly enhanced after IL‐33 treatment.^50^ However, only B1 cells express ST2,^51^, ^52^ so this may also be a marker to distinguish B1 cells from B2 cells. Effector function in B cells after treatment of IL‐33 has been reported. Levels of ST2 expression in B1 cells were found to be robustly elevated.^52^ *In vitro*, IL‐33 has the ability to induce the expression of cytokines, including IL‐5 and IL‐13 secretion.^50^ In addition, IL‐33 can enhance the expression of chemokines and growth factors, primarily including monocyte chemoattractant protein‐1 (MCP‐1), MIP‐1 and VEGF, which induce migration and growth of monocytes/macrophages.^52^

Interestingly, Breg‐like cells were observed following IL‐33 rejection in WT mice. These Breg^IL‐33^ cells can block the development of inflammation in IBD mice by suppressing the pathogenic Th1 responses.^53^ Accordingly, IL‐10‐producing B cells in pericardial adipose tissue of mice were abundantly lower. Analyses of ST2 knockout mice further validated the action of IL‐33.^51^

### Tregs

Certain subsets of Tregs, including those in adipose tissue and intestines, constitutively express high amounts of ST2.^54^, ^55^, ^56^, ^57^ This suggests that these cells act as target of IL‐33. First, IL‐33 is required for the development and maintenance of functional Tregs. Studies have described that IL‐33 promotes the TGF‐β‐mediated Treg differentiation.^54^ In this research, IL‐33 induces the expression of Fopx3, a transcription factor that functions as a master regulator of the Treg phenotype.^54^ It also induces the expression of GATA‐3, which is essential for ST2 expression and stabilises Foxp3 expression, in the form of a feedforward reinforcing manner, and enhances expression.^9^, ^54^ Analysis of ST2^−/−^ Tregs *in vivo* showed Foxp3 expression is deficient.^54^ In another study, combined IL‐33 and IL‐2/STAT5 signals also boost GATA3 expression and therefore promote the Treg development programme.^9^, ^57^ Specifically, the transcription factors IRF4 and BATF are required for the development of Tregs in adipose tissue. IL‐33 promotes the expression of its own receptor by inducing high expression of IRF4 and BATF.^57^

Second, under stimulation of IL‐33, ST2^+^ Tregs exhibit high levels of the activation markers KLRG1, CD103 and OX40.^54^, ^55^, ^58^ They also have potent suppressive capacity,^21^, ^54^ via IL‐10 and AREG production.^55^, ^58^ Third, IL‐33 also boosts the quantity and frequency of Tregs, primarily ST2^+^ Tregs, both *in vitro* and *in vivo*.^54^, ^55^, ^57^, ^58^ However, studies also suggest that this indirect mechanism is mediated by IL‐33 for the expansion of Tregs. This is a result of the IL‐33‐mediated promotion of IL‐2 production by innate immune cells, including DC and MC.^21^, ^24^ It also results from ILC2‐intrinsic IL‐33 signaling,^19^, ^42^ which maintains Treg stability.^21^, ^54^ IL‐33‐ or ST2‐deficient mice showed a serious reduction of Tregs in certain tissues, most notably adipose tissue.^57^ An administration of IL‐33 can reverse this reduction of Tregs and improve tissue damage, suggesting that IL‐33 supports tissue repair, which primarily depends on ST2^+^ Tregs.^21^, ^57^

### CD8^+^ T cells

Only activated CD8^+^ T cells express ST2.^59^, ^60^ Studies have made points to show that primarily CD8^+^ T cells that were generated *in vitro* in the presence of IL‐12 or TCR triggering were reported to express ST2.^59^ IL‐33 augments clonal expansion and antiviral capacity via IFN‐γ production in CD8^+^ T cells.^61^ Specifically, IL‐33 was shown to enhance CD8^+^ T‐cell antigen‐specific responses by expressing CD107a/IFN‐γ in a vaccine setting.^62^ ST2*^−/−^* and IL‐33*^−/−^* mice verified this, which displayed significantly defective CTL responses.^61^

## IL‐33 in host defence

### After virus infection and Th1 immunity

**Figure 1:**
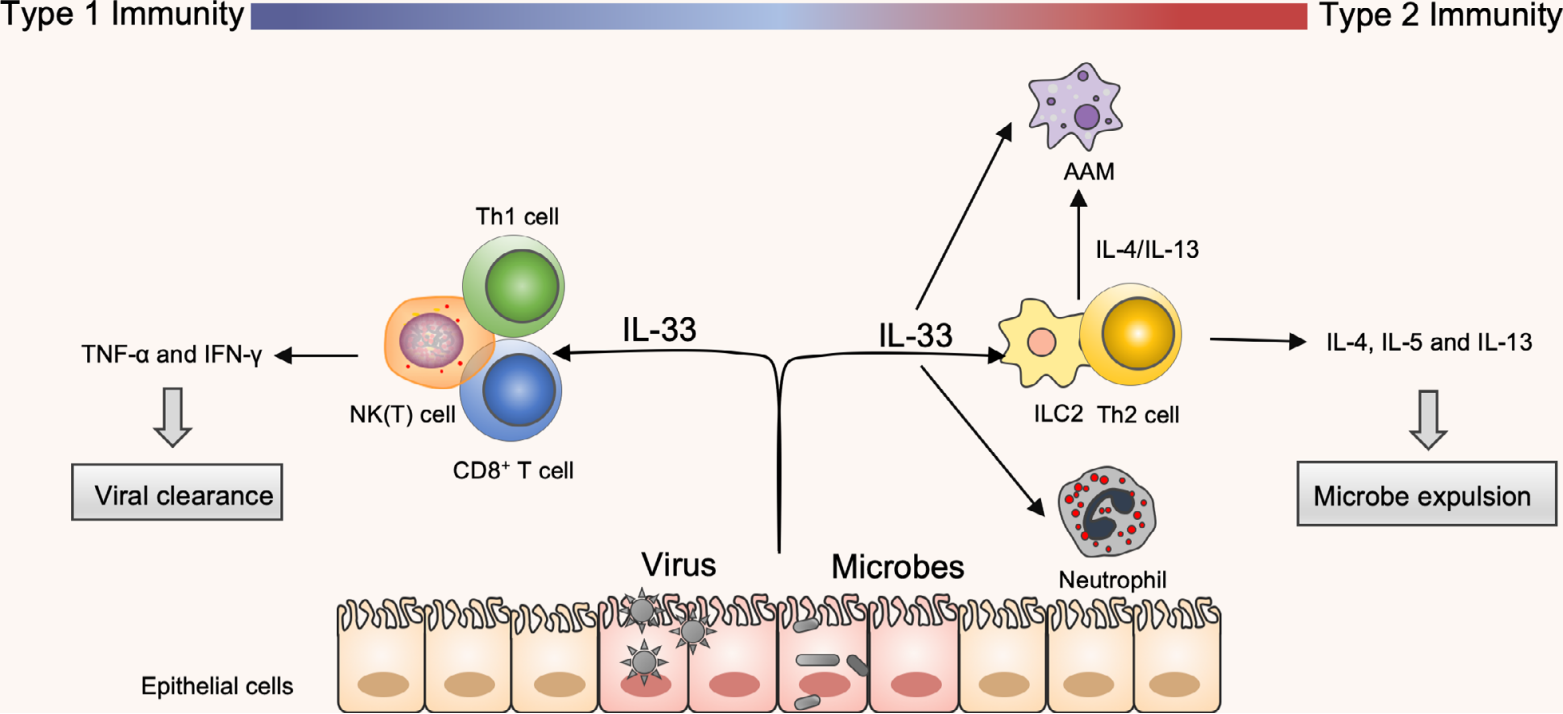
Regulation of distinct modules of IL‐33 in host defence. Type 1 immunity and type 2 immunity are important for the host defence. IL‐33 is expressed by several cell types, but the primary contributors are epithelial cells in barrier tissue. In response to an invading infection, IL‐33 is released from the necrotic cells in order to recruit these immune cells. During a viral infection, released IL‐33 can enhance the function of Th1 cells, NK(T) cells and CD8^+^T cells via TNF‐α and IFN‐γ, thus acting as a protective antiviral response. During microbial infection, IL‐33 activates and expands ILC2s and Th2s, and recruits eosinophils and alternatively activated macrophages (AAMs). These immune cells feedback on the tissue via IL‐4, IL‐5 and IL‐13 and limit microbial infection.

Immune response to viral infections involves Th1 immunity, primarily including CD4^+^ T cells, CD8^+^ T cells and NK cells. They are regarded as the orchestrating effector cells of antiviral immunity. The essential roles and relevant downstream effects of IL‑33 that are important in controlling the reaction to various pathogenic viruses have been studied. The enhanced viral clearance is due to the production of TNF‐α and IFN‐γ. During viral infection, IL‐33 can significantly enhance clonal expansion of ST2^+^CD8^+^T and NK cells to induce a cytotoxic response against a viral load in mice.^13^, ^61^, ^63^ Another important feature of IL‐33 during viral infection is the activation of ST2^+^Th1 cells, which also plays a role in antiviral immunity. Accordingly, IL‐33‐ and ST2‐deficient mice had an increased viral load and developed an exaggerated form of viraemia.^61^ Findings from the Th1 effector cells point to the functional importance of ST2 expression. It shows that the lack of ST2 in CD4^+^ T cells displayed impaired Th1 effector characteristics upon viral infection^11^ (Figure 1).

Moreover, it is now known that IL‐33 is also a crucial amplifier in adaptive immunity by enhancing an antiviral response. By augmenting the accumulation and recall of virus‐specific CD8^+^ memory T cells after infection from vaccinia viruses such as MCMV or LCMV, IL‐33 can promote the production of virus Abs, leading to greater protection against subsequent viral challenge.^60^, ^62^ Taken together, these data demonstrate that IL‐33 might have a role as a vaccine adjuvant to boost viral immunity. The success of this approach might depend on the route of the vaccine and its ability to promote a type 1‐skewed environment. Its exact mechanism needs to be further elucidated.

### After microbial infection and Th2 immunity

The role of IL‐33 in promoting host defence against invading pathogens such as helminths, bacteria or fungi has been extensively studied. Levels of IL‐33 expression were elevated following helminth infection, specifically by *N. brasiliensis* (*Nb*). This infection was accompanied by enhanced Th2 cytokine release and reduced Th1 and Th17 cytokine production.^31^, ^40^ In this context, Th2 cells have been shown to be critical for IL‐33‐dependent infection immunity. In particular, the Th2 cytokine mechanism in ILC2s also confers resistance to microbes, which has been identified as a key role for infection immunity in the absence of adaptive immunity.^35^, ^40^ Systemic IL‐33 can promote pathogen expulsion as a result of the action of ILC2s during sepsis.^31^ In this section, we show that the interactions between ILC2s and Th2 cells act to limit infection and expel pathogens from the site of infection in which they reside.^19^ The protective properties of the IL‐33 during infection can be further enhanced when acting synergistically with other cytokines, including IL‐25.^18^

Another factor in the mechanism by which IL‐33 mediates microbial clearance is through the recruitment of neutrophils and polarisation of AAM. On the one hand, IL‐33 attenuates polymicrobial‐induced sepsis in mice by accelerating microbial clearance and neutrophil recruitment. This is mediated by the elevated levels of phagocytic, fungicidal or bactericidal activity of neutrophils, which are recruited via the production of CXCL1 and CXCL2 by peritoneal macrophages.^30^, ^38^ In addition, the increase in AAM numbers that are seen in IL‐33‐mediated worm killing in the lung^64^ requires CD4^+^ T‐cell‐ and ILC2‐mediated IL‐4/IL‐13 expression^33^, ^35^ (Figure 1). On the other hand, IL‐33 also enhances the antimicrobial activity of dermal macrophages via increased nitric oxide release, thus limiting microbial growth and survival.^65^ This is consistent with the fact that ILC2s and Th2 cells are involved in resistance to infection in an IL‐33‐dependent manner. This is evident as a result of the fact that IL‐33‐deficient mice show a limited elimination of infection. However, the elevated level of IL‐33 can also cause immune suppression via expansion of Tregs, which is commonly observed in sepsis‐surviving patients.^64^ By contrast, IL‐33*^−/−^* mice are more susceptible to infection than wild‐type mice, showing impaired microbial clearance and severe tissue immunopathology.^66^

## IL‐33 in tissue repair

### Immune tolerance and tissue protection

Patients undergoing organ transplantation or experiencing alloimmune disorders could benefit from immune tolerance induction, since dampening inflammatory activation on the target organ is enough to protect tissue. IL‐33 performs specialised functions via immune tolerance induction, and the feasibility of IL‐33 has been tested in acute graft‐versus‐host disease (GVHD) and inflammatory bowel disease (IBD), with promising results.^21^ Data from alloHCT models suggest that IL‐33‐responsive ST2^+^Tregs protect against the alloimmune response that is underlying to GVHD after alloHCT via suppressing macrophage activation and limiting accumulation of effector T cells.^21^ Similar effects are also observed in the intestine. IL‐33 acts in a cell‐intrinsic manner to promote accumulation of ST2^+^ Tregs and prevent dysregulated intestine inflammation, while IL‐23 may disturb this homeostasis and contribute to IBD.^54^ Studies in other mice models indicated that IL‐33‐stimulated Bregs restore colon homeostasis and effectively block the development of spontaneous colitis in IL‐10‐deficient mice.^53^

### Inflammation regulator and tissue restoration

Studies demonstrating several disease models show that IL‐33 induction following injury is associated with improved outcomes. IL‐33 supports tissue repair in several ways. First, IL‐33 limits inflammation by mediating the entry or activation state of certain anti‐inflammation subsets, primarily including AAM and Tregs. They both produce the cytokine IL‐10 in order to control inflammation and restore tissue.^55^ Brain‐derived IL‐33 recruits monocytes and acts on monocytes to augment the differentiation of AAM.^39^ This recruitment, together with a decrease in pro‐inflammatory genes, including IL‐6, IL‐1β and NLRP3, contributes to neuroprotective effects.^39^

**Figure 2:**
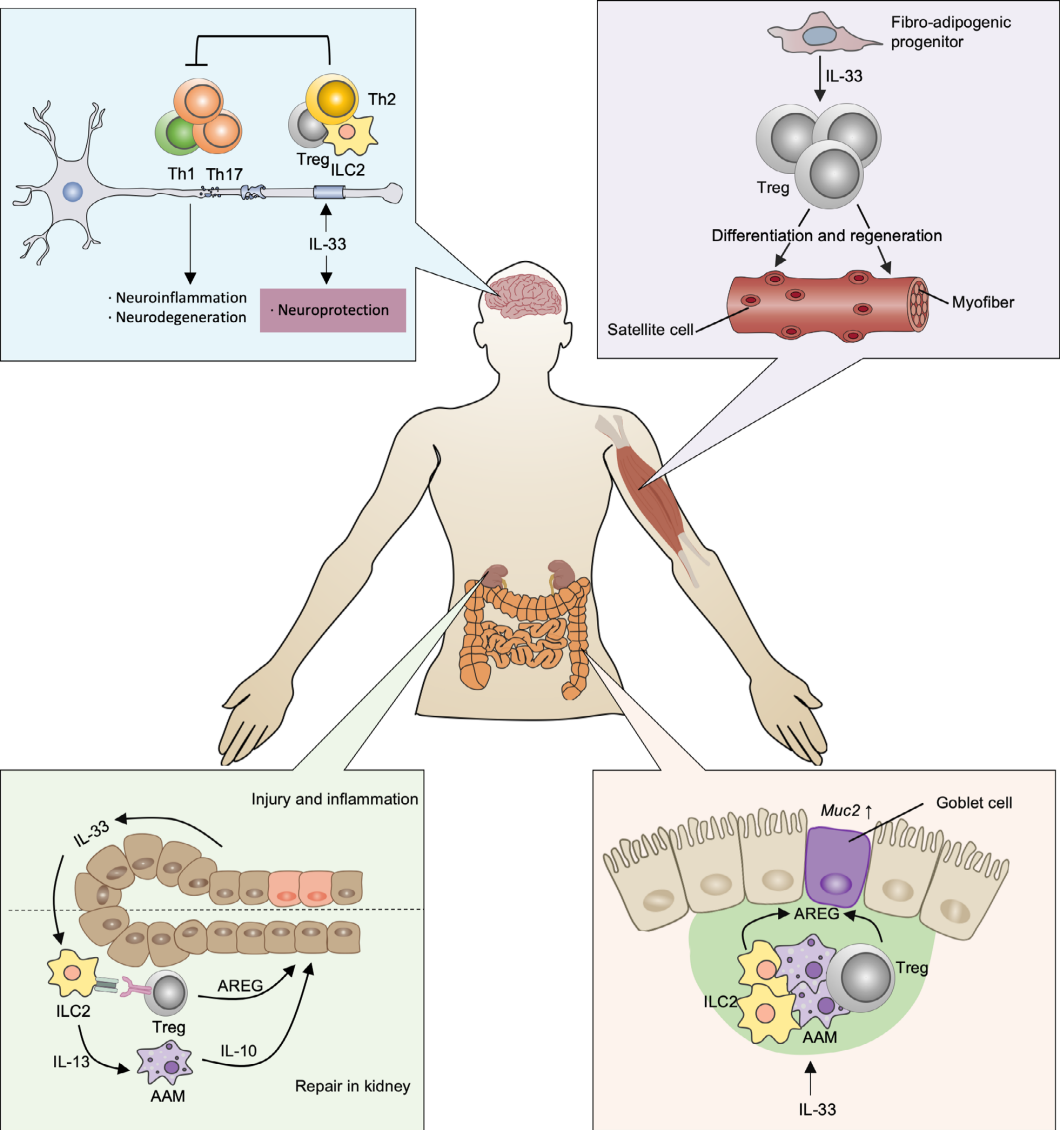
The action modes of IL‐33 during tissue repair and regeneration. IL‐33 confers neuroprotection by stimulating ILC2s and promoting Th2 and Treg accumulation, thereby reversing the pathogenic Th1/Th17 response. Furthermore, IL‐33 activates ILC2s and maintains a type 2 immune environment. These cytokines subsequently induce broad anti‐inflammatory immune cells, such as AAMs and Tregs, that produce IL‐10 and AREG. As a result, the levels of inflammation in the kidney are downgraded and IL‐33 rescues kidney function. In addition, IL‐33 is also important for the regeneration of muscle tissue. It can direct the differentiation and the regeneration of myofibers and satellite cells through Treg expansion. Successful mucosal healing is often achieved by the expression of *Muc* in response to IL‐33, which is present in AREG‐derived ILC2s and Tregs.

Second, IL‐33 maintains a more anti‐inflammatory type 2 cytokine milieu that has evolved to limit and repair tissue damage. There is increasing evidence to link Th2 immunity with the process of tissue restoration. Accordingly, IL‐33 was founded to promote the acceleration of the switch from a pathogenic Th1/Th17 response to a therapeutic Th2‐dominated response in EAE mice^67^ (Figure 2). Further investigations have shown that IL‐33 treatment confers protection by activating ILC2s and reverses established disease.^68^ Similarly, the protective effects of ILC2s have been investigated in models of renal and brain injury.^36^, ^41^, ^69^ Cameron *et al*.^69^ detail evidence suggesting that activation of IL‐33‐stimulated ILC2s and production of a local type 2 immune milieu is protective against injury, particularly in kidney disease. Of note, transfer of IL‐33‐stimulated ILC2s was sufficient to significantly reduce serum creatinine and tubular damage and increase survival following Renal ischaemia–reperfusion injury (IRI), as evidenced by upregulation of the levels of IL‐4 and IL‐13 in serum and kidney.^36^ Interestingly, depletion of ILC2s does not aggravate the severity of injury, suggesting the potential compensation effects from anti‐inflammatory immune cells.^36^, ^70^ In this study, IL‐33‐activated ILC2s were also able to drive AAM and Treg expansion, which is consistent with the mechanism of protection following kidney injury^36^, ^69^ (Figure 2). Collectively, these studies also provide a strong rationale to further explore the therapeutic potential of targeting IL‐33/ILC2 axis in kidney injury.^69^

### Tissue remodelling and regeneration

As outlined earlier, IL‐33 is primarily secreted by inflamed or injured tissue. It preferentially mediates immune cells to promote tissue repair. Specifically, IL‐33 has been shown to have tissue‐regenerative functions in several tissues, including skeletal muscle, skin and the colon. The tissue regeneration function of IL‐33 in the muscles has been attributed in part to its ability to expand ST2^+^Tregs. These Tregs reside and accumulate in the muscle after injury increases the differentiation of satellite cells.^71^ This was the key observation of the supporting role of IL‐33‐primed Tregs in the context of muscle injury. Further, muscle regeneration is compromised by conditional ablation of ST2 genes in Tregs.^56^ A recent study addressed the fact that injection of IL‐33 can restore Treg population and improve muscle regeneration in injured muscles of an aged mouse model. In this study, a shift from the muscle transcriptome towards a muscle regeneration signature was observed, and the number and area of myofibres were also elevated as evidenced by histology.^56^ Upon skin injury, IL‐33‐dependent ILC2 and AAM polarisation promotes cutaneous wound closure and healing by re‐epithelisation and the extracellular matrix (ECM).^72^, ^73^ Accordingly, IL‐33 can also be found to be involved in colon mucosal healing via promoting the differentiation of enterocytes and macrophages into goblet cells and AAM, respectively. The secretory mucin gene *Muc2* and goblet cell hyperplasia gene *KLF* are upregulated,^73^, ^74^ which supports mucosal healing during colitis.

In addition, AREG, a ligand of the epidermal growth factor receptor (EGFR), released by Tregs and ILC2s has important roles in tissue repair of multiple organs. It can induce differentiation and proliferation of its target cells. Emerging studies have shown that the alarmin IL‐33 induces AREG production in ILC2s and Tregs in models of colitis and lung damage after infection. These studies also show that IL‐33 promotes tissue integrity and functional repair^43^, ^58^ (Figure 2). Further, by enhancing the expression of the tight junction protein Claudin 1 and the secretory mucin gene *Muc2*, IL‐33‐stimulated AREG plays a role in tissue repair.^75^ Additional research is needed to further dissect the various tissue‐protective mechanisms of IL‐33 that they induce during the tissue regeneration process.

## IL‐33 in metabolic homeostasis

Metabolic dysfunction is often characterised as systemic low‐grade or a chronic inflammatory state in certain tissues, which is featured as obesity or even as type 2 diabetes. It is indicated that IL‐33 can directly mediate metabolic pathways and hence has a crucial role in metabolic syndrome.

### Metabolic syndrome

Adipose tissue was identified as a reservoir for IL‐33.^76^ Recent observations showed that ST2‐deficient mice were more prone to gain body weight and fat mass, and even disturbances of insulin secretion.^77^, ^78^ Further studies in IL‐33‐deficient mice also demonstrated an impaired glucose tolerance and insulin resistance, even when on a normal diet.^57^, ^78^, ^79^ Importantly, administration of recombinant IL‐33 in obese mice significantly improved multiple metabolic parameters.^78^, ^79^ IL‐33 regulates metabolism by several mechanisms.

First, IL‐33 maintains glucose and insulin homeostasis. Resident type 2 and regulatory immune cells within adipose tissue, including AAM, Tregs, eosinophils and ILC2s, maintain tissue metabolism homeostasis by blocking type 1 immunity, which has been confirmed by other findings. By promoting Treg proliferation and development, IL‐33 dampens obesity‐related inflammation and decreases fasting glucose.^57^, ^79^ In addition, by polarisation of macrophages towards AAM, IL‐33 creates protective effects in obese mice.^77^ Further, ILC2s, AAM and eosinophils constitute a regulatory network under the action of IL‐33. In this network, ILC2s produce IL‐5 and IL‐13 to maintain numbers of eosinophils and phenotype of AAM, respectively. In turn, eosinophils induce IL‐4 to promote AAM expansion.^78^, ^79^, ^80^ Strikingly, a recent study reported that islet mesenchymal cells can produce IL‐33, which directly contributes to insulin secretion. By acting on islet‐resident ILC2s that induce RA‐producing capacities in macrophages and DCs via IL‐13 and colony‐stimulating factor 2, this enhances β‐cell function, and therefore, islet‐derived IL‐33 induces insulin secretion.^5^

**Figure 3:**
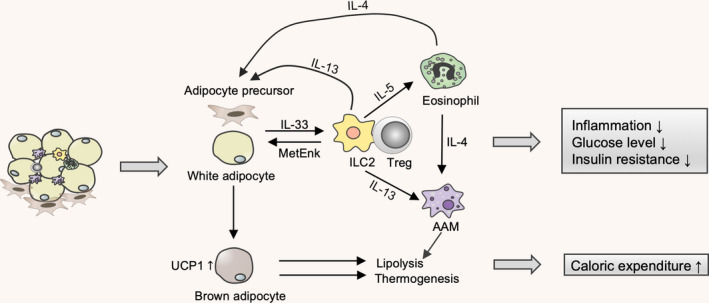
IL‐33 alleviates metabolic syndrome. Mesenchymal cells or adipocyte‐derived IL‐33 in adipose tissue orchestrates an immunometabolic crosstalk and supports and maintains metabolic homeostasis by activating a type 2 immune response. IL‐33 enhances Treg proliferation to achieve protective effects. Further, ILC2s, AAMs and eosinophils can constitute a regulatory network under the action of IL‐33 via IL‐4, IL‐5 and IL‐13 production. Therefore, dampened inflammation, lowered glucose levels and increased insulin sensitivity were observed. In addition, ILC2‐derived MetEnk peptides and AAM can promote lipolysis and thermogenesis for regulating caloric expenditure.

Second, IL‐33 regulates lipid metabolism and energy homeostasis. An emerging cell type that is critical for regulating caloric expenditure is the brown adipocyte. Brown adipose tissue (BAT) expresses high amounts of uncoupling protein 1 (UCP1) and converts energy into heat. On the one hand, IL‐33 treatment can significantly upregulate UCP1 expression in white adipose tissue, which is termed the process of browning of white adipose tissue.^79^, ^81^, ^82^ This occurs via ILC2‐derived methionine‐enkephalin (MetEnk) peptides.^79^ On the other hand, ILC2‐derived IL‐13 and eosinophil‐derived IL‐4 promote proliferation of adipocyte precursors and a beige fate in an IL‐4Rα‐dependent manner.^81^ Particularly, ILC2 activation by IL‐33 favors macrophages towards a protective AAM (Figure 3), which directly leads to a reduced lipid accumulation and gene expression of lipid metabolism and adiposeness, such as *C*/*EBPα* (CAAT enhancer‐binding protein‐α), *SREBP‐1c* (sterol regulatory element‐binding protein 1c), *liver X receptor (LXR)α*, *LXRβ* and *PPARγ*.^77^

### Atherosclerosis

Atherosclerosis is frequently associated with metabolic syndrome. Although IL‐33 is expressed by normal and atherosclerotic vasculature of mice and humans, elevated protective low‐density lipoprotein (oxLDL) antibodies and reduced atherosclerotic plaque size were observed in ApoE‐deficient mice on a high‐fat diet (which serve as mouse models of atherosclerosis).^83^ Because of this respect, IL‐33 directs the immune response towards the Th2 phenotype, which is associated with increased IL‐4, IL‐5, IL‐10 and IL‐13. It also leads to lesser accumulation of macrophages derived from foam cells, as well as T cells.^83^, ^84^, ^85^

## Targeting IL‐33 in human disease

### Allergic inflammation disease

The level of IL‐33 is upregulated in allergic diseases such as asthma,^7^, ^15^, ^86^ atopic dermatitis (AD)^2^, ^87^ and allergic rhinitis.^32^ The concentration of IL‐33 positively correlates with the disease severity.^87^ Studies in animal models that either administer or ablate IL‐33 support its contribution to the pathogenesis of allergic disease.^32^, ^86^ Particularly, *IL‐33* and *ST2* were two major susceptibility loci shown to be associated with human asthma. The role of IL‐33 in asthma has been identified in genome‐wide association studies. Further analyses revealed that the pathogenic role has been ascribed to IL‐33 on the basis of its capacity to initiate innate and adaptive type 2 immunity that is characterised by the production of IL‐4, IL‐5 and IL‐13. IL‐33 administration to mice induces the accumulation and activation of basophils, neutrophils and eosinophils, which results in airway and nasal allergic inflammation.^15^, ^32^ On the one hand, IL‐33 promotes food‐induced anaphylaxis and aggravates allergic reactions through mast cell degranulation.^2^, ^26^ Additionally, IL‐33 also induces ILC2 accumulation, migration and type 2 cytokine production; the release of which induces tissue damage. In this regard, it is well recognised that the IL‐33/ILC2 axis plays a critical role in allergic disease.^7^, ^19^ On the other hand, adaptive Th2 cells are also targets for IL‐33, which are the main drivers of allergy‐associated inflammation once individuals are exposed to the allergen.^19^, ^45^, ^47^ Antibody‐targeting IL‐33 has been proven successful in mouse disease models, with ameliorated symptoms of experimental allergic asthma.^86^ Strikingly, etokimab, an anti‐IL‐33‐humanised monoclonal antibody, has undergone a randomised phase 2a clinical trial in the treatment of patients with moderate‐to‐severe AD and has shown promising efficacy.^88^

### Autoimmunological inflammation disease

The pathogenesis of IL‐33 has been studied extensively in autoimmune diseases, including rheumatoid arthritis (RA), psoriasis and systemic lupus erythematosus (SLE).^12^ In addition, levels of IL‑33 are significantly higher in synovial fluid (SF) and serum from patients with RA and psoriasis than in such samples from healthy donors.^89^, ^90^ In the mouse model of RA, IL‐33 treatment markedly exacerbated arthritis and even erosion of cartilage.^91^, ^92^ The most studied effects of IL‐33 relate to the immune disorder, which were skewed towards a Th1/Th17 phenotype, characterised by the production of TNF‐α, IFN‐γ and IL‐17, as well as autoantibodies. The administration of recombinant IL‐33 markedly upregulated a pro‐inflammatory response, including TNF‐α, IL‐1β, IL‐17 and TNF‐α, synovial hyperplasia, and serum IgG1 and IgG2a in experimentally induced autoimmune arthritis.^37^, ^91^, ^92^ The activation and degranulation of mast cells under action of IL‐33 exacerbates arthritis and skin inflammation.^90^, ^91^, ^92^ IL‐33 also drives neutrophil migration to sites of inflammation in the joints and skin.^37^, ^90^ Moreover, an increased number of ILC2s that produced GM‐CSF in response to IL‐33 are found to initiate and augment arthritis.^93^ In further support for pathogenic functions of IL‐33 cytokines in arthritis, IL‐33 signalling blockade was shown to attenuate experimentally induced autoimmune arthritis.^12^, ^92^

### Cancer progress

A role for IL‐33 in the regulation of carcinogenesis and tumor growth and metastasis has emerged. IL‐33 levels were found to be increased and correlated with tumor development, suggesting that IL‐33 is a potential marker for a poor prognosis.^22^, ^94^, ^95^ Evidence linking IL‐33 to tumor promotion includes preclinical models and human prognostic associations. Three major biological activities of IL‐33 may contribute to poor outcomes with immune‐dependent mechanisms.

First, IL‐33 expression has also been shown to promote premalignant adenoma in the intestines through mast cell‐derived proteases and cytokines.^96^ Second, by mechanisms involving angiogenesis, IL‐33 promotes tumor proliferation. It has been reported that IL‐33 can activate mast cells to produce a chemotactic cytokine, which promotes the accumulation of tumor‐associated macrophages (TAM) and supports the growth of tumor vascular network in gastric cancer.^22^ Last, stromal IL‐33 facilitates tumor metastases and invasion by suppressing local antitumor immunity, which is mediated by intratumor accumulation of Tregs and ILC2s. These cells produce AREG and IL‐13, respectively, and therefore forms a pro‐tumorigenic microenvironment in the breast, lung, head and neck, and colon.^94^, ^97^, ^98^, ^99^

Taken together, targeting IL‐33 remains a therapeutic option for dysfunction and allergic disease. In fact, Clinicaltrials.gov has listed anti‐IL‐33 clinical trials in patients with asthma, food allergy, chronic rhinosinusitis and chronic obstructive airway disease (accessed 19 February 2020). The findings from these clinical trials confirm their potential therapeutic value by means of blocking their immunological effects in IL‐33‐neutralising antibodies. The need to explore IL‐33‐targeting therapeutic strategies and possibilities to maximise their clinical efficacy remains, even though results are impressive.

## Concluding remarks

The recent studies we have discussed within this paper suggest that IL‐33 is a key immune orchestrator. These ST2‐expressing immune cells are involved in a complex dialogue with IL‐33. Through interaction with other immune cells, IL‑33 amplifies inflammatory responses and contributes to immune pathology, especially during allergic and autoimmune inflammation. There are few clinical trials for the treatment of these diseases via blockade of IL‐33 in progress. Furthermore, IL‐33 also has unique roles in driving tissue protection and regeneration, as well as homeostasis. These unique properties need to be carefully considered before utilising an IL‑33 cytokine therapy, suggesting that more research is required. However, many questions remain unsolved. Although studies have shown that the processing of the full‐length form of IL‐33 into biologically active forms is a crucial regulatory mechanism in IL‐33 biology, the question of how both the full‐length and truncated forms of IL‐33 function *in vivo* remains unknown. Emerging data have been obtained using mouse models, but more human clinical trials may be required to answer whether IL‐33 will be an available therapy to treat human diseases. Most importantly, a recent report identified an IL‐33 expression mesenchymal cell in adipose tissue, thus termed as IL‐33 reservoir. Upon inflammatory stimuli, the release of IL‐33 could promote anti‐inflammatory effects and maintain tissue homeostasis. However, it is uncertain whether or not this repair role was also observed in other diseases. Studies from the mesenchymal stem cell (MSC) field point to the fact that only inflammatory licensed MSCs engage in therapeutic actions through a series of immune cells. Whether allograft MSCs produce IL‐33 during the transfer process and contribute to tissue repair in an IL‐33‐dependent manner remains unexplained and requires further exploration.

## Author contributions

**Zekun Zhou:** Conceptualization; Visualization; Writing‐original draft. **Fei Yan:** Writing‐review & editing. **Ousheng Liu:** Conceptualization; Funding acquisition; Supervision.

## Conflict of interest

The authors declare no conflict of interest.

## References

[1] Schmitz J , Owyang A , Oldham E *et al* IL‐33, an interleukin‐1‐like cytokine that signals via the IL‐1 receptor‐related protein ST2 and induces T helper type 2‐associated cytokines. Immunity 2005; 23: 479–490. 16286016 10.1016/j.immuni.2005.09.015

[2] Leyva‐Castillo JM , Galand C , Kam C *et al* Mechanical skin injury promotes food anaphylaxis by driving intestinal mast cell expansion. Immunity 2019; 50: 1262–1275. 31027995 10.1016/j.immuni.2019.03.023 PMC6531322

[3] Martin NT , Martin MU . Interleukin 33 is a guardian of barriers and a local alarmin. Nat Immunol 2016; 17: 122–131. 26784265 10.1038/ni.3370

[4] Ferhat M , Robin A , Giraud S *et al* Endogenous IL‐33 contributes to kidney ischemia‐reperfusion injury as an alarmin. J Am Soc Nephrol 2018; 29: 1272–1288. 29436517 10.1681/ASN.2017060650 PMC5875946

[5] Dalmas E , Lehmann FM , Dror E *et al* Interleukin‐33‐activated islet‐resident innate lymphoid cells promote insulin secretion through myeloid cell retinoic acid production. Immunity 2017; 47: 928–942. 29166590 10.1016/j.immuni.2017.10.015

[6] Cayrol C , Duval A , Schmitt P *et al* Environmental allergens induce allergic inflammation through proteolytic maturation of IL‐33. Nat Immunol 2018; 19: 375–385. 29556000 10.1038/s41590-018-0067-5

[7] Li Y , Chen S , Chi Y *et al* Kinetics of the accumulation of group 2 innate lymphoid cells in IL‐33‐induced and IL‐25‐induced murine models of asthma: a potential role for the chemokine CXCL16. Cell Mol Immunol 2019; 16: 75–86. 30467418 10.1038/s41423-018-0182-0 PMC6318283

[8] Cayrol C , Girard J‐P . IL‐33: an alarmin cytokine with crucial roles in innate immunity, inflammation and allergy. Curr Opin Immunol 2014; 31: 31–37. 25278425 10.1016/j.coi.2014.09.004

[9] Peine M , Marek RM , Löhning M . IL‐33 in T cell differentiation, function, and immune homeostasis. Trends Immunol 2016; 37: 321–333. 27055914 10.1016/j.it.2016.03.007

[10] Kearley J , Silver Jonathan S , Sanden C *et al* Cigarette smoke silences innate lymphoid cell function and facilitates an exacerbated type I interleukin‐33‐dependent response to infection. Immunity 2015; 42: 566–579. 25786179 10.1016/j.immuni.2015.02.011

[11] Baumann C , Bonilla WV , Fröhlich A *et al* T‐bet– and STAT4–dependent IL‐33 receptor expression directly promotes antiviral Th1 cell responses. Proc Natl Acad Sci USA 2015; 112: 4056–4061. 25829541 10.1073/pnas.1418549112 PMC4386370

[12] Liew FY , Girard J‐P , Turnquist HR . Interleukin‐33 in health and disease. Nat Rev Immunol 2016; 16: 676. 27640624 10.1038/nri.2016.95

[13] Nabekura T , Girard JP , Lanier LL . IL‐33 receptor ST2 amplifies the expansion of NK cells and enhances host defense during mouse cytomegalovirus infection. J Immunol 2015; 194: 5948–5952. 25926677 10.4049/jimmunol.1500424 PMC4458425

[14] Göpfert C , Andreas N , Weber F *et al* The p38‐MK2/3 module is critical for IL‐33–induced signaling and cytokine production in dendritic cells. J Immunol 2018; 200: 1198–1206. 29288203 10.4049/jimmunol.1700727

[15] Sun B , Zhu L , Tao Y *et al* Characterization and allergic role of IL‐33‐induced neutrophil polarization. Cell Mol Immunol 2018; 15: 782–793. 29503441 10.1038/cmi.2017.163 PMC6141612

[16] de Kleer IM , Kool M , de Bruijn MJW *et al* Perinatal activation of the interleukin‐33 pathway promotes type 2 immunity in the developing lung. Immunity 2016; 45: 1285–1298. 27939673 10.1016/j.immuni.2016.10.031

[17] Nobs SP , Natali S , Pohlmeier L *et al* PPARγ in dendritic cells and T cells drives pathogenic type‐2 effector responses in lung inflammation. J Exp Med 2017; 214: 3015–3035. 28798029 10.1084/jem.20162069 PMC5626395

[18] Huang Y , Guo L , Qiu J *et al* IL‐25‐responsive, lineage‐negative KLRG1^hi^ cells are multipotential 'inflammatory' type 2 innate lymphoid cells. Nat Immunol 2015; 16: 161–169. 25531830 10.1038/ni.3078 PMC4297567

[19] Halim TYF , Rana BMJ , Walker JA *et al* Tissue‐restricted adaptive type 2 immunity is orchestrated by expression of the costimulatory molecule OX40L on group 2 innate lymphoid cells. Immunity 2018; 48: 1195–1207. 29907525 10.1016/j.immuni.2018.05.003 PMC6015114

[20] Oh JE , Oh DS , Jung HE , Lee HK . A mechanism for the induction of type 2 immune responses by a protease allergen in the genital tract. Proc Natl Acad Sci USA 2017; 114: E1188–E1195. 28137851 10.1073/pnas.1612997114 PMC5320955

[21] Matta BM , Reichenbach DK , Zhang X *et al* Peri‐alloHCT IL‐33 administration expands recipient T‐regulatory cells that protect mice against acute GVHD. Blood 2016; 128: 427–439. 27222477 10.1182/blood-2015-12-684142 PMC4957164

[22] Eissmann MF , Dijkstra C , Jarnicki A *et al* IL‐33‐mediated mast cell activation promotes gastric cancer through macrophage mobilization. Nat Commun 2019; 10: 2735. 31227713 10.1038/s41467-019-10676-1 PMC6588585

[23] Emi‐Sugie M , Toyama S , Matsuda A , Saito H , Matsumoto K . IL‐33 induces functional CCR7 expression in human mast cells. J Allergy Clin Immunol 2018; 142: 1341–1344. 29928923 10.1016/j.jaci.2018.06.007

[24] Morita H , Arae K , Unno H *et al* An interleukin‐33‐Mast cell‐interleukin‐2 axis suppresses papain‐induced allergic inflammation by promoting regulatory T cell numbers. Immunity 2015; 43: 175–186. 26200013 10.1016/j.immuni.2015.06.021 PMC4533925

[25] Joulia R , L'Faqihi F‐E , Valitutti S , Espinosa E . IL‐33 fine tunes mast cell degranulation and chemokine production at the single‐cell level. J Allergy Clin Immunol 2017; 140: 497–509. 27876627 10.1016/j.jaci.2016.09.049

[26] Sjöberg LC , Gregory JA , Dahlén SE , Nilsson GP , Adner M . Interleukin‐33 exacerbates allergic bronchoconstriction in the mice via activation of mast cells. Allergy 2015; 70: 514–521. 25660244 10.1111/all.12590

[27] Wang JX , Kaieda S , Ameri S *et al* IL‐33/ST2 axis promotes mast cell survival via BCLXL. Proc Natl Acad Sci USA 2014; 111: 10281–10286. 24982172 10.1073/pnas.1404182111 PMC4104908

[28] Cherry WB , Yoon J , Bartemes KR , Iijima K , Kita H . A novel IL‐1 family cytokine, IL‐33, potently activates human eosinophils. J Allergy Clin Immunol 2008; 121: 1484–1490. 18539196 10.1016/j.jaci.2008.04.005 PMC2821937

[29] Cohen M , Giladi A , Gorki A‐D *et al* Lung single‐cell signaling interaction map reveals basophil role in macrophage imprinting. Cell 2018; 175: 1031–1044. 30318149 10.1016/j.cell.2018.09.009

[30] Alves‐Filho JC , Sônego F , Souto FO *et al* Interleukin‐33 attenuates sepsis by enhancing neutrophil influx to the site of infection. Nat Med 2010; 16: 708–712. 20473304 10.1038/nm.2156

[31] Krishack PA , Louviere TJ , Decker TS *et al* Protection against Staphylococcus aureus bacteremia‐induced mortality depends on ILC2s and eosinophils. JCI Insight 2019; 4: e124168. 10.1172/jci.insight.124168 PMC6482999 30721149

[32] Haenuki Y , Matsushita K , Futatsugi‐Yumikura S *et al* A critical role of IL‐33 in experimental allergic rhinitis. J Allergy Clin Immunol 2012; 130: 184–194 e111. 22460070 10.1016/j.jaci.2012.02.013

[33] Tran VG , Kim HJ , Kim J *et al* IL‐33 enhances host tolerance to candida albicans kidney infections through induction of IL‐13 production by CD4^+^ T cells. J Immunol 2015; 194: 4871–4879. 25847973 10.4049/jimmunol.1402986

[34] Espinassous Q , Garcia‐de‐Paco E , Garcia‐Verdugo I *et al* IL‐33 enhances lipopolysaccharide‐induced inflammatory cytokine production from mouse macrophages by regulating lipopolysaccharide receptor complex. J Immunol 2009; 183: 1446–1455. 19553541 10.4049/jimmunol.0803067

[35] Bouchery T , Kyle R , Camberis M *et al* ILC2s and T cells cooperate to ensure maintenance of M2 macrophages for lung immunity against hookworms. Nat Commun 2015; 6: 1–13. 10.1038/ncomms7970 25912172

[36] Cao Q , Wang Y , Niu Z *et al* Potentiating tissue‐resident type 2 innate lymphoid cells by IL‐33 to prevent renal ischemia‐reperfusion injury. J Am Soc Nephrol 2018; 29: 961–976. 29295873 10.1681/ASN.2017070774 PMC5827602

[37] Verri WA Jr , Souto FO , Vieira SM *et al* IL‐33 induces neutrophil migration in rheumatoid arthritis and is a target of anti‐TNF therapy. Ann Rheum Dis 2010; 69: 1697–1703. 20472598 10.1136/ard.2009.122655

[38] Li D , Guabiraba R , Besnard A‐G *et al* IL‐33 promotes ST2‐dependent lung fibrosis by the induction of alternatively activated macrophages and innate lymphoid cells in mice. J Allergy Clin Immunol 2014; 134: 1422–1432. 24985397 10.1016/j.jaci.2014.05.011 PMC4258609

[39] Fu AK , Hung KW , Yuen MY *et al* IL‐33 ameliorates Alzheimer's disease‐like pathology and cognitive decline. Proc Natl Acad Sci USA 2016; 113: E2705–2713. 27091974 10.1073/pnas.1604032113 PMC4868478

[40] Frisbee AL , Saleh MM , Young MK *et al* IL‐33 drives group 2 innate lymphoid cell‐mediated protection during Clostridium difficile infection. Nat Commun 2019; 10: 2712. 31221971 10.1038/s41467-019-10733-9 PMC6586630

[41] Gadani SP , Smirnov I , Smith AT , Overall CC , Kipnis J . Characterization of meningeal type 2 innate lymphocytes and their response to CNS injury. J Exp Med 2017; 214: 285–296. 27994070 10.1084/jem.20161982 PMC5294864

[42] Molofsky Ari B , Van Gool F , Liang H‐E *et al* Interleukin‐33 and interferon‐γ counter‐regulate group 2 innate lymphoid cell activation during immune perturbation. Immunity 2015; 43: 161–174. 26092469 10.1016/j.immuni.2015.05.019 PMC4512852

[43] Monticelli LA , Osborne LC , Noti M *et al* IL‐33 promotes an innate immune pathway of intestinal tissue protection dependent on amphiregulin–EGFR interactions. Proc Natl Acad Sci USA 2015; 112: 10762–10767. 26243875 10.1073/pnas.1509070112 PMC4553775

[44] Christian S , Adnan RK , Achilleas F *et al* ILC2s regulate adaptive Th2 cell functions via PD‐L1 checkpoint control. J Exp Med 2017; 214: 2507–2521. 28747424 10.1084/jem.20170051 PMC5584124

[45] Guo L , Huang Y , Chen X *et al* Innate immunological function of TH2 cells *in vivo* . Nat Immunol 2015; 16: 1051–1059. 26322482 10.1038/ni.3244 PMC4575627

[46] Piehler D , Grahnert A , Eschke M *et al* T1/ST2 promotes T helper 2 cell activation and polyfunctionality in bronchopulmonary mycosis. Mucosal Immunol 2012; 6: 405–414. 22990621 10.1038/mi.2012.84

[47] Ting C , Christopher AT , Xiaogang F *et al* PPAR‐γ promotes type 2 immune responses in allergy and nematode infection. Sci Immunol 2017; 2: eaal5196. 28783701 10.1126/sciimmunol.aal5196

[48] Pascual‐Reguant A , Bayat Sarmadi J , Baumann C *et al* TH17 cells express ST2 and are controlled by the alarmin IL‐33 in the small intestine. Mucosal Immunol 2017; 10: 1431–1442. 28198366 10.1038/mi.2017.5

[49] Komai‐Koma M , Wang E , Kurowska‐Stolarska M *et al* Interleukin‐33 promoting Th1 lymphocyte differentiation dependents on IL‐12. Immunobiology 2016; 221: 412–417. 26688508 10.1016/j.imbio.2015.11.013 PMC4731778

[50] Komai‐Koma M , Gilchrist DS , McKenzie AN *et al* IL‐33 activates B1 cells and exacerbates contact sensitivity. J Immunol 2011; 186: 2584–2591. 21239718 10.4049/jimmunol.1002103

[51] Wu L , Dalal R , Cao CD *et al* IL‐10‐producing B cells are enriched in murine pericardial adipose tissues and ameliorate the outcome of acute myocardial infarction. Proc Natl Acad Sci USA 2019; 116: 21673–21684. 31591231 10.1073/pnas.1911464116 PMC6815157

[52] Ahmed A , Koma MK . Interleukin‐33 triggers B1 cell expansion and its release of monocyte/macrophage chemoattractants and growth factors. Scand J Immunol 2015; 82: 118–124. 25997709 10.1111/sji.12312

[53] Sattler S , Ling G‐S , Xu D *et al* IL‐10‐producing regulatory B cells induced by IL‐33 (Breg^IL‐33^) effectively attenuate mucosal inflammatory responses in the gut. J Autoimmun 2014; 50: 107–122. 24491821 10.1016/j.jaut.2014.01.032 PMC4012142

[54] Schiering C , Krausgruber T , Chomka A *et al* The alarmin IL‐33 promotes regulatory T‐cell function in the intestine. Nature 2014; 513: 564–568. 25043027 10.1038/nature13577 PMC4339042

[55] Ito M , Komai K , Mise‐Omata S *et al* Brain regulatory T cells suppress astrogliosis and potentiate neurological recovery. Nature 2019; 565: 246–250. 30602786 10.1038/s41586-018-0824-5

[56] Kuswanto W , Burzyn D , Panduro M *et al* Poor repair of skeletal muscle in aging mice reflects a defect in local, interleukin‐33‐dependent accumulation of regulatory T cells. Immunity 2016; 44: 355–367. 26872699 10.1016/j.immuni.2016.01.009 PMC4764071

[57] Vasanthakumar A , Moro K , Xin A *et al* The transcriptional regulators IRF4, BATF and IL‐33 orchestrate development and maintenance of adipose tissue‐resident regulatory T cells. Nat Immunol 2015; 16: 276–285. 25599561 10.1038/ni.3085

[58] Arpaia N , Green JA , Moltedo B *et al* A distinct function of regulatory T cells in tissue protection. Cell 2015; 162: 1078–1089. 26317471 10.1016/j.cell.2015.08.021 PMC4603556

[59] Ngoi SM , St Rose MC , Menoret AM *et al* Presensitizing with a Toll‐like receptor 3 ligand impairs CD8 T‐cell effector differentiation and IL‐33 responsiveness. Proc Natl Acad Sci USA 2012; 109: 10486–10491. 22689946 10.1073/pnas.1202607109 PMC3387033

[60] Baumann C , Frohlich A , Brunner TM *et al* Memory CD8^+^ T cell protection from viral reinfection depends on interleukin‐33 alarmin signals. Front Immunol 2019; 10: 1833. 31447845 10.3389/fimmu.2019.01833 PMC6692449

[61] Bonilla WV , Fröhlich A , Senn K *et al* The alarmin interleukin‐33 drives protective antiviral CD8^+^ T cell responses. Science 2012; 335: 984–989. 22323740 10.1126/science.1215418

[62] McLaren JE , Clement M , Marsden M *et al* IL‐33 augments virus‐specific memory T cell inflation and potentiates the efficacy of an attenuated cytomegalovirus‐based vaccine. J Immunol 2019; 202: 943–955. 30635396 10.4049/jimmunol.1701757 PMC6341181

[63] Sesti‐Costa R , Silva GK , Proenca‐Modena JL *et al* The IL‐33/ST2 pathway controls coxsackievirus B5‐induced experimental pancreatitis. J Immunol 2013; 191: 283–292. 23733876 10.4049/jimmunol.1202806

[64] Nascimento DC , Melo PH , Pineros AR *et al* IL‐33 contributes to sepsis‐induced long‐term immunosuppression by expanding the regulatory T cell population. Nat Commun 2017; 8: 1–14. 28374774 10.1038/ncomms14919 PMC5382289

[65] Prince A , Li C , Li H *et al* Interleukin‐33 increases antibacterial defense by activation of inducible nitric oxide synthase in skin. PLoS Pathog 2014; 10. 10.1371/journal.ppat.1003918 PMC3930573 24586149

[66] Mahapatro M , Foersch S , Hefele M *et al* Programming of intestinal epithelial differentiation by IL‐33 derived from pericryptal fibroblasts in response to systemic infection. Cell Rep 2016; 15: 1743–1756. 27184849 10.1016/j.celrep.2016.04.049

[67] Jiang HR , Milovanovic M , Allan D *et al* IL‐33 attenuates EAE by suppressing IL‐17 and IFN‐γ production and inducing alternatively activated macrophages. Eur J Immunol 2012; 42: 1804–1814. 22585447 10.1002/eji.201141947

[68] Russi AE , Ebel ME , Yang Y , Brown MA . Male‐specific IL‐33 expression regulates sex‐dimorphic EAE susceptibility. Proc Natl Acad Sci USA 2018; 115: E1520–E1529. 29378942 10.1073/pnas.1710401115 PMC5816140

[69] Cameron GJM , Jiang SH , Loering S *et al* Emerging therapeutic potential of group 2 innate lymphoid cells in acute kidney injury. J Pathol 2019; 248: 9–15. 30684265 10.1002/path.5242

[70] Cameron GJM , Cautivo KM , Loering S *et al* Group 2 innate lymphoid cells are redundant in experimental renal ischemia‐reperfusion injury. Front Immunol 2019; 10: 826. 31057549 10.3389/fimmu.2019.00826 PMC6477147

[71] Burzyn D , Kuswanto W , Kolodin D *et al* A special population of regulatory T cells potentiates muscle repair. Cell 2013; 155: 1282–1295. 24315098 10.1016/j.cell.2013.10.054 PMC3894749

[72] Rak GD , Osborne LC , Siracusa MC *et al* IL‐33‐dependent group 2 innate lymphoid cells promote cutaneous wound healing. J Invest Dermatol 2016; 136: 487–496. 26802241 10.1038/JID.2015.406 PMC4731037

[73] Seo DH , Che X , Kwak MS *et al* Interleukin‐33 regulates intestinal inflammation by modulating macrophages in inflammatory bowel disease. Sci Rep 2017; 7: 851. 28404987 10.1038/s41598-017-00840-2 PMC5429815

[74] Waddell A , Vallance JE , Moore PD *et al* IL‐33 signaling protects from murine oxazolone colitis by supporting intestinal epithelial function. Inflamm Bowel Dis 2015; 21: 2737–2746. 26313694 10.1097/MIB.0000000000000532 PMC4654638

[75] Kolodin D , van Panhuys N , Li C *et al* Antigen‐ and cytokine‐driven accumulation of regulatory T cells in visceral adipose tissue of lean mice. Cell Metab 2015; 21: 543–557. 25863247 10.1016/j.cmet.2015.03.005 PMC4747251

[76] Rana BMJ , Jou E , Barlow JL *et al* A stromal cell niche sustains ILC2‐mediated type‐2 conditioning in adipose tissue. J Exp Med 2019; 216: 1999–2009. 31248899 10.1084/jem.20190689 PMC6719433

[77] Miller AM , Asquith DL , Hueber AJ *et al* Interleukin‐33 induces protective effects in adipose tissue inflammation during obesity in mice. Circ Res 2010; 107: 650–658. 20634488 10.1161/CIRCRESAHA.110.218867 PMC4254700

[78] Mahlakõiv T , Flamar A‐L , Johnston L *et al* Stromal cells maintain immune cell homeostasis in adipose tissue via production of interleukin‐33. Sci Immunol 2019; 4: eaax0416. 31053655 10.1126/sciimmunol.aax0416 PMC6766755

[79] Brestoff JR , Kim BS , Saenz SA *et al* Group 2 innate lymphoid cells promote beiging of white adipose tissue and limit obesity. Nature 2015; 519: 242–246. 25533952 10.1038/nature14115 PMC4447235

[80] Chang SK , Kohlgruber AC , Mizoguchi F *et al* Stromal cell cadherin‐11 regulates adipose tissue inflammation and diabetes. J Clin Invest 2017; 127: 3300–3312. 28758901 10.1172/JCI86881 PMC5669565

[81] Lee MW , Odegaard JI , Mukundan L *et al* Activated type 2 innate lymphoid cells regulate beige fat biogenesis. Cell 2015; 160: 74–87. 25543153 10.1016/j.cell.2014.12.011 PMC4297518

[82] Odegaard JI , Lee MW , Sogawa Y *et al* Perinatal licensing of thermogenesis by IL‐33 and ST2. Cell 2016; 166: 841–854. 27453471 10.1016/j.cell.2016.06.040 PMC4985267

[83] Miller AM , Xu D , Asquith DL *et al* IL‐33 reduces the development of atherosclerosis. J Exp Med 2008; 205: 339–346. 18268038 10.1084/jem.20071868 PMC2271006

[84] McLaren JE , Michael DR , Salter RC *et al* IL‐33 reduces macrophage foam cell formation. J Immunol 2010; 185: 1222–1229. 20543107 10.4049/jimmunol.1000520

[85] Zhang H‐F , Wu M‐X , Lin Y‐Q *et al* IL‐33 promotes IL‐10 production in macrophages: a role for IL‐33 in macrophage foam cell formation. Exp Mol Med 2017; 49: e388. 29099095 10.1038/emm.2017.183 PMC5704190

[86] Allinne J , Scott G , Lim WK *et al* IL‐33 blockade affects mediators of persistence and exacerbation in a model of chronic airway inflammation. J Allergy Clin Immunol 2019; 144: 1624–1637. 31562870 10.1016/j.jaci.2019.08.039

[87] Tamagawa‐Mineoka R , Okuzawa Y , Masuda K , Katoh N . Increased serum levels of interleukin 33 in patients with atopic dermatitis. J Am Acad Dermatol 2014; 70: 882–888. 24569114 10.1016/j.jaad.2014.01.867

[88] Chen Y‐L , Gutowska‐Owsiak D , Hardman C *et al* Proof‐of‐concept clinical trial of etokimab shows a key role for IL‐33 in atopic dermatitis pathogenesis. Sci Transl Med 2019; 11: eaax2945. 31645451 10.1126/scitranslmed.aax2945

[89] Yang Z , Liang Y , Xi W , Li C , Zhong R . Association of increased serum IL‐33 levels with clinical and laboratory characteristics of systemic lupus erythematosus in Chinese population. Clin Exp Med 2011; 11: 75–80. 20963466 10.1007/s10238-010-0115-4

[90] Hueber AJ , Alves‐Filho JC , Asquith DL *et al* IL‐33 induces skin inflammation with mast cell and neutrophil activation. Eur J Immunol 2011; 41: 2229–2237. 21674479 10.1002/eji.201041360

[91] Xu D , Jiang HR , Li Y *et al* IL‐33 exacerbates autoantibody‐induced arthritis. J Immunol 2010; 184: 2620–2626. 20139274 10.4049/jimmunol.0902685

[92] Xu D , Jiang HR , Kewin P *et al* IL‐33 exacerbates antigen‐induced arthritis by activating mast cells. Proc Natl Acad Sci USA 2008; 105: 10913–10918. 18667700 10.1073/pnas.0801898105 PMC2491487

[93] Hirota K , Hashimoto M , Ito Y *et al* Autoimmune Th17 cells induced synovial stromal and innate lymphoid cell secretion of the cytokine GM‐CSF to initiate and augment autoimmune arthritis. Immunity 2018; 48: 1220–1232. 29802020 10.1016/j.immuni.2018.04.009 PMC6024031

[94] Wen YH , Lin HQ , Li H *et al* Stromal interleukin‐33 promotes regulatory T cell‐mediated immunosuppression in head and neck squamous cell carcinoma and correlates with poor prognosis. Cancer Immunol Immunother 2019; 68: 221–232. 30357458 10.1007/s00262-018-2265-2 PMC11028137

[95] Yue Y , Lian J , Wang T *et al* Interleukin‐33‐nuclear factor‐κB‐CCL2 signaling pathway promotes progression of esophageal squamous cell carcinoma by directing regulatory T cells. Cancer Sci 2020; 111: 795–806. 31883400 10.1111/cas.14293 PMC7060484

[96] Maywald RL , Doerner SK , Pastorelli L *et al* IL‐33 activates tumor stroma to promote intestinal polyposis. Proc Natl Acad Sci USA 2015; 112: E2487–E2496. 25918379 10.1073/pnas.1422445112 PMC4434739

[97] Halvorsen EC , Franks SE , Wadsworth BJ *et al* IL‐33 increases ST2^+^ Tregs and promotes metastatic tumour growth in the lungs in an amphiregulin‐dependent manner. Oncoimmunology 2019; 8: e1527497. 30713780 10.1080/2162402X.2018.1527497 PMC6343789

[98] Pastille E , Wasmer M‐H , Adamczyk A *et al* The IL‐33/ST2 pathway shapes the regulatory T cell phenotype to promote intestinal cancer. Mucosal Immunol 2019; 12: 990–1003. 31165767 10.1038/s41385-019-0176-y PMC7746527

[99] Kudo‐Saito C , Miyamoto T , Imazeki H *et al* IL33 is a key driver of treatment resistance of cancer. Cancer Res 2020; 80: 1981–1990. 32156776 10.1158/0008-5472.CAN-19-2235

